# Digital Economy and Green Development: Mechanisms of Action, Spillover Effects and Transmission Mechanisms

**DOI:** 10.3390/e27090966

**Published:** 2025-09-17

**Authors:** Xin Tong, Ke Li, Xuesen Li

**Affiliations:** 1 School of Business Administration, Northeastern University, Shenyang 110819, China; 2 College of Science and Technology, Shenyang Polytechnic College, Shenyang 110021, China

**Keywords:** digital economy, green development, role mechanism, spillover effect, transmission mechanism

## Abstract

The digital economy plays an important role in promoting green economic growth. This study evaluates the degree of green economic development generated by green innovation and green sharing based on data from 30 provinces in China from 2011 to 2022. An empirical analysis of the digital economy’s influence on the growth of the green economy and its transmission mechanisms is performed. The analysis results demonstrate that the digital economy can significantly promote green economic development, encompassing improvements in both green innovation and green sharing, and exhibits a nonlinear “increasing marginal effect”. The analysis of transmission channels reveals that, on one hand, the digital economy can promote green economic development by optimizing the allocation of data elements, while on the other, its impact is also influenced by the intensity of environmental regulations, exhibiting a threshold effect. Further heterogeneity analysis suggests that the promotional effect of the digital economy on green economic development is more pronounced in regions with high levels of economic development, a robust infrastructure, and strong policy support.

## 1. Introduction

The digital economy, as a new form of economy, has played an important role in promoting green development in countries around the world. The European Union focus on promoting green development through the digital economy is mainly on how digital technology can help achieve green transformation goals, optimizing the allocation and efficient utilization of renewable energy through smart grids, energy management systems, and other means. Applying digital technology to supply and demand management of industrial energy users to improve industrial process efficiency and achieve wiser decision-making. The European Union is promoting a dual transformation of green and digital through industrial clusters, with German companies accounting for 45% of total research and development expenditures in their respective regions. Over the past five years, they have applied for more than 200 green energy patents. The United States attaches great importance to the integration and application of digital technology and net zero emissions targets. China has elevated digital empowerment of green transformation to a national strategy and actively promoted comprehensive green transformation of economic and social development, which is a major strategic decision made by the Party Central Committee.

Achieving green economic development remains among the key aims of China’s high-quality economic growth strategy. The digital economy is the central trend of global modernization at present, and the integration of rapidly advancing technology may aid China’s sustainable growth. Through the use of big data, the blockchain, and cloud computing, the digital economy can facilitate the digital transformation of industries, giving birth to new industrial models and increasing energy efficiency [1]. Meanwhile, the digital economy has also become deeply ingrained in all spheres of life and work, significantly contributing to the efficiency of green innovation and the greening of various industries [2]. Hence, it is practically significant to assess the impact of the digital economy on the growth of the green economy and to identify the transmission channels required to create a high-quality economy.

Current scholarship on the digital economy and green economic growth comprises three areas, including the notion of the internet-based economy and its measurement. The term “digital economy” first emerged in the 1996 book *Digital Economy* by the American scholar Don Tapscott, and due to the continued advancement of digital technology, domestic and international academics hold diverse viewpoints on this concept. Early international studies defined it as digital-technology-related production processes [3,4], while it was later expanded to include commercial activities that use the Internet to exchange products and services with the advancement of ICT [5,6]. Domestic academics have investigated its significance from various perspectives. For instance, some have pointed out that digital technology and other tools are primarily responsible for producing the digital economy [7], while several academics have noted that the economy that describes the digital flow is called the digital economy [8]. According to some researchers, the main component of the digital economy is data; hence, commercial activities that can fully utilize digital technology are referred to as being part of the digital economy [9]. Several investigators have split the digital economy into two or three categories, including the information industry, the information technology and equipment industry, and the data value-added industry [10,11]. The term “digital economy” has therefore had many different definitions, both domestically and internationally, and a unified consensus has not yet been reached. In addition, there is also little agreement on how to quantify the digital economy: the academic community mainly divides digital economy measurement indicators into single-indicator systems and multi-dimensional indicator systems, with single indicators mainly including postal and telecommunications business volume, computer and Internet penetration rate, etc. [12,13,14], and multi-dimensional indicators mainly relating to information technology infrastructure, information network access, and information technology effects [15,16,17].

Another important area of research in this field is the meaning, significance, and influence of green economic development. The British academic David Pearce initially introduced the idea of the “green economy” in 1989 [18]. Subsequently, studies conducted domestically and abroad have discussed green economic development from many perspectives. Foreign scholars have pointed out that green development should adhere to the principles of sustainable development and at the same time respect ecological limitations, while domestic scholars have argued that the green economy should be developed in terms of sustainability and coordination [19,20,21]. Academics mainly divide the measurement methods for green economic development into two types, with the first being founded on the conventional DEA model, which primarily gauges the degree of green economic development by considering resources, capital, and labor as inputs and ecological and environmental pollution as undesirable outputs [22,23,24], and the second primarily employing PCA, the coefficient of variation, and entropy TOPSIS methods to assess the state of green economic growth [25,26,27,28]. The majority of scientists have focused on the effects of the degree of environmental regulation, industrial structure, energy structure, science and technology level, and the extent of openness to the outside world when analyzing impact factors [29,30,31,32].

Research on the relationship between the digital economy and green economic development is still primarily qualitative, mainly exploring the potential benefits of the digital economy, which has created a new economic structure using the Internet, big data, and developing technology, enhancing production and transaction efficiency and thus supporting long-term economic growth [33,34]. Quantitative approaches are less common, and most of the literature focuses on the impact the digital economy on green production and green ecology [35,36,37], while substantially less is known about the economic elements of green growth.

Combing the existing literature, it is clear that research on the digital economy and green economic development has achieved certain accomplishments. However, most of the literature overlooks the transmission channels through which the digital economy influences green economic development, as well as the nonlinear characteristics of this relationship. This paper therefore contributes to the literature in the following ways: firstly, from the perspectives of green innovation and green sharing, we explore the nonlinear characteristics of the digital economy’s impact on green economic development; and secondly, we select the level of data element allocation as the mediating variable affecting green economic development and use environmental regulation as the threshold variable to comprehensively examine the mechanism of the digital economy’s impact on green development.

## 2. Theoretical Analysis and Research Hypothesis

### 2.1. The Direct Impact of the Digital Economy on the Green Development of the Economy

Although the world has changed dramatically and positively with industrial development, mechanized production is accompanied by the high consumption of resources. Due to the fast growth of Internet technology and the rise in ICT-based services in recent decades, a “new economy” has formed that places a greater emphasis on novel activities and products in the digital economy [38]. Being highly technological and having low environmental costs, it is distinct from the conventional model for economic growth [39], and it has a beneficial effect on economic expansion because it can reduce transaction costs by implementing its own information and intelligent production model using digital technology, as well as because it can reduce marginal production costs and boost resource allocation efficiency by selecting the most suitable producers and production factors through a Hadoop search. It has also a unique educational impact, as regions can learn technology and knowledge from each other, thus driving their own technological progress and green innovation, thereby reducing environmental costs and improving the environment. Consequentially, the digital economy is unquestionably in line with the ideas of “resource-saving” and “environmental friendliness”, providing support for coordinating economic growth and promoting the ecological environment. As a result, the following hypothesis is proposed.

**H1.**  *The digital economy has a direct contribution to the green development of the economy.*

### 2.2. The “Nonlinear” Spillover Effect of the Digital Economy on Green Economic Development

From the perspective of input and output, diverse industrial agglomeration makes information pooling easier, which leads to the agglomeration of industries with small differences, strong correlations, and unique characteristics, which is conducive to inter-industry communication, cooperation, and mutual learning. This effect is reinforced when using digital technology, leading to green technology innovation and improvements in production efficiency, thus promoting local economic development and achieving the unification of economic and ecological benefits. Additionally, because the value of a network is equal to the square of its nodes [40], as the digital economy develops, primarily through the supply of network technology, its significance grows, and the economic activities of various sectors can overcome the limitations of distance, while different economic agents can participate in the same economic activities in different spaces. This makes the exchange of economic activities and talent and the flow of capital between different regions more convenient, which is more conducive to reducing transaction costs, realizing green technological innovation, achieving economies of scale, and reducing environmental burdens. As a result, the following hypothesis is put forth.

**H2.**  *The digital economy has a nonlinear “increasing marginal effect” on the green development of the economy.*

### 2.3. Analysis of the Transmission Mechanisms of the Digital Economy on the Green Development of the Economy

With the emergence of the information era, the definition of factors of production is no longer based on the traditional aspects of land, capital, labor, etc. Data, a fundamental component, has the same standing as capital and labor, and its production attributes are continuously improving. The asymmetry of information in the traditional production line often leads to the distortion of resource allocation, inefficiencies in production, and the massive consumption of energy, which in turn leads to the destruction of the ecological environment. In contrast, the high mobility and permeability that are intrinsic to data can successfully address the issue of information inequality [41], thus maximizing the distribution of data resources based on the knowledge available, minimizing resource allocation distortion, and enhancing energy efficiency. The digital economy can best allocate data factors through two main routes: first, its high permeability can efficiently and precisely match production factors, encourage the deep integration of data factors with traditional factors, and then bring the maximum value of data factors into play, and second, its high mobility offers a platform for the concentration and flow of data factors, which aids in the effective allocation and flow of data factors. Thus, the subsequent hypothesis is suggested.

**H3.**  *The digital economy can promote the green development of the economy by optimizing the allocation of data elements.*

Green economic development is not only influenced by the digital economy, but also by environmental regulations. Based on an in-depth study of “Porter’s hypothesis”, it was found that environmental regulation and the green economy no longer have a straightforward linear connection; instead, they have a nonlinear one, displaying a “positive U-shape” [42] or an “inverted U-shape” [43]. In addition, evidence suggests that the growth of the digital economy might mitigate the detrimental effects of environmental regulations on green TFP [44]. Hence, it is beneficial to look at the degree of environmental regulation to better comprehend the link between the development of the green economy and the digital economy. Low standards for environmental regulation result in areas paying less attention to environmental regulatory regulations, and as economies become more intelligent and digital, economic actors are more concerned with financial gains than ecological gains. Economic agents must take environmental costs into account as the level of environmental regulatory oversight rises in order to increase profits. As the digital economy develops, economic agents will be able to fully utilize big data and other computing infrastructures to lower production costs, encourage the development of green technologies, and lower the costs associated with emission reduction, resulting in the convergence of economic and ecological benefits. The environmental costs that economic agents must bear significantly rise as the extent of environmental regulation increases. This results in higher production costs and fewer competitive advantages. Currently, economic agents use digital technology to find more economically advantageous economic activities in order to avoid a high intensity of environmental control, which lessens the role of the digital economy in accelerating green economic development, Therefore, the following hypotheses are proposed in this study.

**H4.**  *The impact of the digital economy on the green development of the economy is also influenced by the intensity of environmental regulations, which exhibits a threshold effect.*

## 3. Data Processing and Model Design

### 3.1. Variable Selection and Data Sources

(1) Explained variables

The level of green economic development (*Enc*) is the explained variable in this article. The core goal of green development is to achieve a coordinated unity of economic growth, resource conservation, environmental improvement, and social equity, rather than a single dimension of environmental protection or emission reduction. Regarding the construction of indicators for green economic development, this study specifically measures green economic development from two aspects: green innovation and green sharing. Green innovation solves the technical and efficiency issues of how to achieve green transformation, while green sharing solves the fairness and inclusiveness issues of who to achieve green transformation for. The indicators of green innovation and green sharing, respectively, respond to the driving force and value destination of green development, and together constitute a complete logical loop from feasibility to sustainability of green development. Therefore, they are key dimensions for measuring green development.

In order to ensure the rationality of the indicator system, this study follows the principles of comprehensiveness, scientificity, representativeness, and operability in constructing the evaluation indicator system. The selection of green innovation indicators is essentially a precise anchoring of the driving force and practical feasibility of green development, integrating sustainable development concepts into the innovation process, and achieving coordinated progress between economic development and environmental protection through the development and application of new technologies, products, and services. Therefore, in this study, green innovation is examined from three perspectives: innovation environment, innovation input, and innovation output, while green sharing is evaluated from two aspects: green production and green living. The innovation environment is evaluated based on three aspects: human capital index, number of R&D personnel, and technology market turnover rate, from the perspectives of theoretical adaptability, practical necessity, and data availability; Examine innovation investment from three perspectives: research and development expenditure, technology expenditure, proportion of local general public budget expenditure, and proportion of environmental protection expenditure to fiscal expenditure; Examine innovation output from two perspectives: the number of granted invention patents and the proportion of sales revenue of new products in high-tech industries to GDP; Examine green production from three aspects: total investment in environmental pollution control, output value of new products from industrial enterprises above designated size, and electricity consumption per unit of regional GDP; We examined green living from three perspectives: per capita daily water consumption, green coverage rate in built-up areas, and harmless treatment rate of household waste.

The selection of green sharing indicators is essentially a precise definition of the value destination of green development, transforming the green concept of a community with a shared future for mankind into quantifiable and assessable practical standards, and providing a fair and shared Chinese model for global green governance. Therefore, according to the selection criteria of green sharing indicators, the core is to return to the fairness and inclusiveness essence of green development, focusing on the collaborative cooperation of various entities such as government, enterprises, social organizations, and the public, achieving the organic unity of resource conservation, environmental protection, and social equity, allowing all people to share the fruits of green development, and integrating the development model of green development with the concept of shared development. Green development is closely related to the quality of life of residents. In urban planning, increasing the construction of green spaces and parks provides residents with more leisure and entertainment space. At the same time, green spaces play a regulating role in urban climate, alleviate urban heat island effect, improve residents’ quality of life, and enable residents to live in a more comfortable and healthy environment. Therefore, green sharing is examined from two aspects: green production and green living, specifically including waste utilization, sewage treatment, green construction, urban environment, and social security, which are measured in three levels of indicators. Table 1 lists the specific classifications of indicators, with indicator properties “+” or “−”representing their effectiveness. This article also uses the entropy weight method to measure the degree of green economic development which is widely used to objectively calculate the weights of indicators.

(2) Explanatory variables

The explanatory variable used in this study is the digital economy’s degree of development (*Dig*). As a unique means of production, the digital economy relies on data as its key production factor. The utilization of horizontal data is a distinctive production factor that distinguishes the digital economy from traditional economies. Therefore, the efficiency of its collection, circulation, utilization, and security directly determines the quality of digital economy development. In order to fully reflect the scientific connotation of digital economy development level, this article constructs a quantifiable and systematic evaluation framework for digital economy development from five aspects: digital economy development scale, digital technology innovation, digital industry development, digital finance, and digital economy development potential. At the same time, the development level of the digital economy has multidimensional, cross disciplinary, and dynamic characteristics, covering both hard support such as infrastructure and technological innovation, as well as soft empowerment such as industrial integration and application penetration, and basic guarantees such as institutional environment and security. According to the indicator measurement standards and the availability of data, as shown in Table 2, and indicator properties “+” representing their effectiveness. This article also adopts the entropy weight method to measure the development level of the digital economy.

(3) Intermediate variables

Data factor allocation (*Ele*) is selected as the mediating variable in this study. The Fourth Plenary Session of the Nineteenth Central Committee clearly put forward that “data can participate in value distribution as a factor of production”, and the digital economy mainly influences economic development through the allocation of data factors. As an intangible factor of production, data factor allocation cannot be directly measured by a single indicator. Therefore, this study draws on the analytical approaches of other scholars to measure the level of data factor allocation based on four dimensions: data element management, data development applications, data dissemination and sharing, and the data application environment, and further refine the indicator system by combining the characteristics of data elements in different dimensions of measurement standards and data availability, The specific categorization of indicators is shown in Table 3.

(4) Control variables

The degree of government intervention (*Gov*), the level of urbanization (*Urban*), human capital investment (*Rz*), the level of infrastructure (*Lnf*), and the level of openness (*Fdi*) are chosen as the control variables in this study based on research conducted by Huang Zhelin and other scholars [45]. *Gov* is defined as the proportion of local general government spending to *GDP*; Urban is reflected in the rate of urbanization; *Rz* is calculated as the proportion of R&D employees to the overall population; *Lnf* is given as the share of total post and telecommunications business volume in GDP; and *Fdi* is calculated as the share of regional foreign direct investment in GDP.

(5) Data sources

In this work, data from 30 provinces in China from 2011 to 2022 were used as the research sample. The Xizang Autonomous Region was excluded due to the availability of data, and missing values were supplemented by means of linear interpolation. The data mainly came from the China Statistical Yearbook, provincial statistical yearbooks, the China High-Tech Industry Statistical Yearbook, and the China Rural Statistical Yearbook, as well as the Atmospheric Composition Analysis Group of Dalhousie University in Canada, the Guotai An Database, and the CEE Database. Table 4 presents the descriptive statistical results of the variables in this article.

### 3.2. Model Setting

(1) Baseline regression model

The benchmark regression model that this study builds to examine the direct influence of the digital economy on the green development of the economy is created in order to validate the aforementioned research hypothesis.

(1) $$Enc_{it}=\alpha_{0}+\beta_{0}Dig_{it}+\beta_{1}X_{it}+\mu_{i}+\delta_{t}+\varepsilon_{\mathrm{it}}$$

where i is the province; t is the year; Enc_it_ is the indicator of the green economic development level; Dig_it_ is the indicator of the digital economy’s degree of development; α_0_ is the constant; β_0_ is the regression coefficient; X_it_ is the control variable; μ_it_ is the province fixed effect; δ_it_ is the year fixed effect; and ɛ_it_ is the random disturbance term.

(2) Transmission channel test

In order to discuss the possible mechanisms of the effects of the digital economy on the green development of the economy, this paper first selects the data factor allocation (Ele) as the mediating variable and builds a model based on Formula (1) by referring to the mediating effect analysis method proposed by other scholars [46], which is as follows:

(2) $$E{\mathrm{le}}_{\mathrm{it}}=\varphi_{0}+\varphi_{1}Dig_{it}+\varphi_{2}X_{it}+\mu_{i}+\delta_{t}+\varepsilon_{it}$$

(3) $$Enc_{it}=\gamma_{0}+\gamma_{1}Dig_{it}+\gamma_{2}Ele_{it}+\gamma_{3}X_{it}+\mu_{i}+\delta_{t}+\varepsilon_{it}$$

In addition to the mediating effect model, a panel threshold model can be used to further explore the nonlinear spillover effect. This study refers to the research of scholars such as Liu Rongzeng and selects the degree of environmental regulation as the threshold variable [47], where the degree of environmental regulation is characterized by the proportion of completed investment in industrial pollution control to secondary industry value added, and the specific panel threshold model is structured as follows:

(4) $$Enc_{it}=\alpha_{0}+\beta_{1}D{\mathrm{ig}}_{\mathrm{it}}•I(Th_{it}\leq \theta )+\beta_{3}Dig_{it}•I(Th_{it}>\theta )+\beta_{2}X_{it}+\mu_{i}+\delta_{t}+\varepsilon_{it}$$

where Th_it_ is the threshold variable; θ is the threshold value; I (•) is the indicator function for segmentation according to different thresholds; and the meanings of the other letters correspond to those in Equation (1). Equation (4) considers only the single-threshold case, but can be expanded to multiple-threshold cases depending on the actual sample.

## 4. Analysis of Results

### 4.1. Baseline Regression Results

The initial regression findings for the impact of the digital economy on the growth of the green economy are displayed in Table 5. This study employs a progressive regression methodology, where models (1) and (2) only control for time and individuals, whereas models (3) and (4) also incorporate control variables based on time- and individual-level control, respectively. The findings demonstrate that although the correlation coefficients of models (3) and (4) decrease after the addition of control variables, the overall effect of the digital economy on green innovation and green sharing is significantly positive at the 1% confidence level, meaning that the digital economy directly contributes to the development of the green economic sector, supporting Hypothesis 1 of this paper. The level of urbanization has a positive impact on green sharing, but it is not significant. The reason for this may be that as the level of urbanization increases, people’s demands for quality of life also rise, and green living gradually becomes a requirement for a high-quality life. Therefore, improving the level of urbanization can also promote green economic development. However, in reality, factors such as imperfect sharing mechanisms, limited indicator systems, significant regional differences, and lagging institutional cultures lead to its impact on green development being insignificant. From Table 5, investing in human capital has a favorable impact on both green innovation and green sharing, and at the 1% confidence level, this impact is highly beneficial. Labor is the most basic factor of production for all production activities, and in the context of the current era, General Secretary Xi Jinping pointed out that talented employees are the future, meaning that human capital investment can promote green innovation, green sharing, and thus green economic development. The level of infrastructure has a negative impact on green innovation and green sharing, with a greater impact on green sharing. The reason for this may be that the infrastructure system has systemic defects in green orientation, structural adaptation, and sharing mechanisms, exerting a reverse inhibitory effect on green development to some extent. Therefore, infrastructure urgently needs to be transformed into “green infrastructure” and “shared platforms”. Both green innovation and green sharing are positively impacted by the degree of external openness; however, this effect is only substantial for green sharing. The focus on the green economy is not as prominent as it should be, and this could be because external openness is currently focused mostly on improving economic status and quality of life.

Since the impact of the digital economy on green economic development has a lagging effect, this study further lags the digital economy by two to three periods. Table 6 presents the specific outcomes, demonstrating that when the lag period is two to three periods, it has a significant incremental effect on green innovation, and this effect decreases as the number of lag periods increases, while it also has a crucial dynamic incremental influence on green sharing, with this effect increasing as the number of lag periods increases. This dynamic superposition impact on green sharing is primarily due to the availability of digital technology, which allows various economic organizations to interact across regions and sectors. In addition to providing a stage for factor movement and data element agglomeration, its high mobility also fosters ecological innovation.

### 4.2. Robustness Test and Endogeneity Treatment

This study employs both the tailing procedure for validation and the replacement of the estimation technique of the key explanatory variables to evaluate the robustness of the findings. The entropy weight technique was used in the prior empirical study to estimate the main explanatory factors. This study further uses the principal component analysis technique to remeasure the key explanatory factors in order to guarantee the robustness of the experimental findings. The results of the regression are given in Table 7, which demonstrates that the digital economy is still significantly positive for green innovation and green sharing. Additionally, since the data used in this study may contain outliers, a 1% tail-reduction treatment was applied to the major variables in order to further reduce the likelihood that outliers will affect the empirical findings. Table 7 displays the outcomes, which still have a sizable favorable impact. In conclusion, Hypothesis 1 remains true regardless of how the key explanatory factors are estimated or whether the tail treatment is shrunk. As a consequence, the empirical findings presented in this article are trustworthy.

Since the core explanatory variables may be endogenous, this paper further conducts the Hausman test, and the results reject the original hypothesis with probability *p* = 0, indicating that the core explanatory variables are endogenous explanatory variables. Therefore, endogeneity is treated using the instrumental variable technique in this article, which uses the overall telecommunications business volume as the instrumental variable. Firstly, the widespread availability of the Internet is primarily responsible for the growth of the digital economy, and as the industry that underpins Internet expansion, telecommunications is intimately linked to the development of the digital economy. Secondly, telecommunication business, which is mainly realized through traditional telecommunication tools, has little impact on the green development of the economy and satisfies the exclusivity requirements. Table 8 lists the statistical results, which show that the first stage F values are all higher than 10, no weak instrumental variables were selected, and the impact of the digital economy on green innovation and green sharing is significantly positive, demonstrating the reliability of the empirical findings presented here.

### 4.3. Analysis of Transmission Channels

Previous experiments have demonstrated a direct boost to green economic growth by the digital economy. This study undertakes a transmission channel analysis in the hope of better comprehending the digital economy’s indirect influence, with data factor allocation being used as the mediating variable. Table 8 illustrates the predictive outcomes for the mediating model. The coefficient estimates of models (1) and (2) show that Hypothesis 1 is accurate, while model (3) demonstrates that the digital economy also has noteworthy influences on data factors. Data factor configuration has a mediating influence, as evidenced by model (4)’s significant positive coefficient for the impact of data element allocation on green innovation, which is 0.665. Meanwhile, the correlation between the digital economy and green innovation in model (4) is 0.118, which is significant too, referring to the theoretical analysis of causal inference performed by Jiang Ting [48], indicating that the data factor allocation has a full mediating effect. The regression result of model (5) verifies whether data factor allocation has a mediating effect between digital economy and green sharing, and the regression coefficient for the impact of data factor allocation on green sharing is 1.134 and significant, indicating that data factor allocation has a mediating effect; in addition, the regression coefficient for the impact of the digital economy on green sharing in model (5) is positive and insignificant, consequently suggesting that the data factor configuration completely mediates the relationship. In conclusion, Hypothesis 3 is true and the digital economy can support green economic development through the optimization of data factor allocation.

In order to further investigate the transmission mechanisms of the digital economy influencing green economic development, this article tests whether a threshold effect exists by using the digital economy and environmental regulation as threshold variables, respectively. The following results were derived by using Stata 16 software to repeat the sample 1000 times. Table 9 exhibits that when the digital economy is as the threshold variable, it significantly passes the single-threshold test, where the threshold value is the same in the regressions with green innovation and green sharing as the explanatory variables, at 0.3797. When environmental regulation is used as the threshold variable, it passes the single-threshold test, whether the dependent variable is green innovation or green sharing, being 0.1173 and 0.3479, respectively. Table 10 presents the regression results with digital economy and environmental regulation as threshold variables. The following results are obtained after controlling for the degree of government intervention, urbanization level, human capital investment, infrastructure level, and level of openness to the outside world.

The regression coefficients when the threshold variable is the digital economy are 0.096 and 0.979 and substantially positive when Th < 0.3797; the regression coefficients when Th > 0.3797 are 0.228 and 0.382, respectively, and pass the test at the 5% confidence level. It is clear that as the digital economy continues to grow, so does its promoting effect on the degree of green economic development, suggesting that it has a nonlinear “increasing marginal effect” and that Hypothesis 2 is true.

The regression coefficients for the impact of the digital economy on green innovation and green sharing are notably positive when environmental regulation is used as the threshold variable. When green innovation is used as the explanatory variable, the regression results are 0.096, 0.228, and 0.339, respectively, all of which meet the requirements at a 1% degree of confidence, while when green sharing is used as the explanatory variable, the regression outcomes are 0.979, 0.382, and 0.509, respectively, with all passing the test at a 5% degree of confidence. This study thus comes to the conclusion that Hypothesis 4 is true and that the severity of environmental restrictions has a threshold effect on how the digital economy affects green economic development.

## 5. Further Analysis: Heterogeneity Analysis

The previous sections of this article established that the digital economy contributes to green economic development. However, the following question still remains: does the digital economy have varied effects on green economic development in different locations and policy contexts? The influence of the digital economy on green economic growth is thus further explored in this section from the perspectives of several regional and policy contexts.

### 5.1. Regional Heterogeneity Analysis

This study splits all areas of China into the eastern, central, and western regions for regression, since different regions have varying levels of infrastructural and economic development. Table 11 shows the regression results. The outcomes demonstrate that while the digital economy has varying degrees of influence on green economic growth in various places, on the whole, it has a major positive effect. In particular, the eastern area has the biggest regression coefficients for the contribution of the digital economy to green economic development (0.867 and 1.209, respectively), demonstrating a greater impact than the central and western regions. The regression coefficients in the central area are 0.178 and 0.130, indicating a significant positive effect on green innovation. Although the overall impact of the digital economy on green economic development is slightly weaker in the western region, the difference between the two is not significant. In the western region, the digital economy only has a significant impact on green sharing, with no significant effect on green innovation. The reasons for these differences may be as follows: first, the degree of economic growth in each region varies greatly, with the eastern and central areas having a higher degree of economic growth than the western region and having progressively developed scale effects. The potential for green innovation and sharing has substantially increased, which is meaningful for the growth of a green economy. Traditional industries have dominated the growth of the western region, and a green economy mindset has not yet taken root. With reliable Internet, AI, and other resources, the eastern and central regions can fully utilize science and technology and contribute to the green economy. However, due to its geographical location, the western region’s infrastructure is not yet complete, and the provision of basic necessities for life mainly depends on pertinent government policies, such as the Western Development Policy, to strengthen major infrastructure construction in the western region. As a result, the western area still has a long way to go in terms of developing its digital economy and green economy.

### 5.2. Policy Heterogeneity Analysis

Since the digital economy is an emerging industry, its development mainly relies on the support of relevant policies, so this study selected the “Belt and Road” initiative proposed in 2013 and the “Action Plan for Promoting the Development of Big Data” proposed in 2015 to investigate the effect of the digital economy on the economy’s green development based on heterogeneity analysis. Firstly, the sample is split into along and non-along areas based on whether they are in the provinces along the “Belt and Road” initiative. Secondly, the “Action Plan for Promoting Big Data Development” issued by the Chinese government in 2015 proposes establishing “comprehensive experiments on big data” in Beijing, Guizhou, and other locations, and thus this article splits the sample into pilot regions and non-pilot areas according to whether “big data comprehensive experiments” are established, with the regression findings being displayed in Table 12. Specifically, under the Belt and Road Initiative, the regression coefficients for the impact of the digital economy on green innovation and green sharing in the regions along the Belt and Road are 0.434 and 0.566, respectively, and are significantly positive. The regression coefficients for the impact of the digital economy on green innovation and green sharing in regions not along the Belt and Road are 0.428 and 0.604, respectively, and are also significantly positive. This indicates that the impact of the digital economy on green innovation and development in the regions along the Belt and Road is higher than that in regions not along the Belt and Road. The Belt and Road Initiative has strengthened the promoting effect of the digital economy on green economic development. Under the policy influence of the “Action Plan for Promoting the Development of Big Data”, it can be found from Table 12 that the regression coefficients for the impact of the digital economy on green innovation and green sharing in pilot regions are 0.474 and 0.902, respectively, while those for non-pilot regions are 0.142 and 0.095, respectively. The regression coefficients in pilot regions are significantly higher than those in non-pilot regions, indicating that the impact of the digital economy on green economic development in pilot regions is significantly higher than that in non-pilot regions. Although the “Action Plan” has improved the digitalization level in pilot regions, due to the gradual implementation of policies, the lengthy transmission chain of green innovation, and the siphoning effect between regions, the differences in green innovation and sharing between pilot and non-pilot regions are not significant. As policies are further deepened and implemented, the promoting effect of the digital economy will gradually become apparent.

## 6. Conclusions and Recommendations

### 6.1. Conclusions

To address the issue of how the digital economy affects green economic development, this article attempts to provide a new explanation based on two perspectives, namely green innovation and green sharing, and its conclusions are as follows: First, the digital economy has a direct promoting effect on green economic development, including green innovation and green sharing, with this “increasing marginal effect” being nonlinear. Second, in terms of transmission channels, on the one hand, optimal data factor allocation has a substantial impact on the digital economy’s impact on green economic growth, while data factor allocation plays a complete intermediary role; on the other hand, the role played by the digital economy in advancing green economic development is positively affected by appropriate environmental legislation, while environmental regulations that are either too weak or too strong will not support the digital economy’s promotion of green economic development. Third, when analyzing heterogeneity, the boost provided by the digital economy to green economic development is more pronounced in areas with a greater degree of infrastructure quality and economic growth, such as eastern and central regions, and it is also more evident in areas with more robust governmental assistance, such as along regions and pilot regions. The findings of this paper thus provide interesting insights into the development of the digital economy and green economic development.

### 6.2. Recommendations

Various provinces in China have made certain achievements in promoting green development through the digital economy. Eastern coastal provinces with strong digital economy foundations need to make breakthroughs in green technology innovation, industrial integration, and digital environmental governance. Central and western provinces, such as Qinghai and Chongqing, are actively exploring green computing power and industrial green transformation with their own resources and policy advantages. Promote overall green development in China through practice and provide diverse paths for sustainable development in various regions. China should continuously develop the digital economy and drive the green development of the economy. Firstly, to encourage the development of the digital economy, China should energetically support projects related to building data infrastructure based on big data and 5G technology and strengthening this infrastructure’s construction to ensure data security. Secondly, it should create a platform for exchange between the digital and green economies, reshape green innovation and sharing paradigms, encourage the growth of green economic activities through networking and intelligence, and create a digital platform for the green development of the economy. China should also maximize the distribution of its data components and reasonably formulate the intensity of its environmental regulations. China should promptly formulate a system for the flow of data factors in green economic activities, the deep blending of data factors with traditional production factors, the maximization of the allocation level of data factors, the enhancement of the utilization level of data factors, and thus the promotion of green economic development. In addition, only reasonable environmental regulations are helpful for promoting the digital economy and green economic growth. China should therefore assess local economic development and set environmental regulations at a reasonable intensity to realize the coexistence of the “silver mountain” and “green mountain”.

China should also put into practice diverse development strategies based on local circumstances and encourage the comprehensive integration of the digital economy and green economic growth. Although the overall economic situation in China is on an upward trend, the degree of economic growth in different areas continues to vary greatly. Leading regions in the east should steadily improve their own levels of digital economic development and enhance coordination between digital and green economic growth. In addition, while focusing on their own construction, the eastern regions should also actively build cooperation and exchange platforms with central and western regions, while these regions to learn from the digital economy development experience of eastern regions and integrate regional elements to achieve a green transition within their economy in accordance with regional circumstances. At the same time, government departments should also implement differentiated development strategies, formulate relevant digitalization and green economic policies, especially by providing digital hardware, and drive the digital economy of less developed regions, so as to release the “digital dividend” of green innovation in these areas. In addition, it is essential to proactively direct the flow of digital capital and scientific and technological talent to underdeveloped areas to promote regional digital innovation and green innovation capabilities.

Many countries have formed their own unique models in promoting green development through the digital economy. The European Green Deal is a policy framework proposed by the European Commission to address climate change, aimed at promoting sustainable development through measures such as clean energy transition and circular economy development. At the level of industrial policy, the EU will focus its development efforts on clean energy, circular economy, digital technology, and other areas. Policy measures cover almost all economic sectors such as industry, agriculture, transportation, and energy to accelerate the transition of the EU economy from a traditional model to a sustainable development model. The goal is to achieve carbon neutrality in the European region by 2050. Denmark and Germany, with their typicality and complementarity in industrial coverage, technological innovation, and policy mechanisms, have become representative countries in the field of promoting green development through digital transformation. Their experience not only fully reflects the mainstream international trend but also highly aligns with the core needs of China’s digital green transformation, providing a “precise, systematic, and operable” reference path for China’s digital green transformation, with the characteristics of “targeted and practical”. Drawing on international practical experience, China should actively develop advanced digital technologies refer to Denmark’s experience in the “application scenario landing” of digital technologies, solve the problem of “difficult landing and poor effectiveness” of digital green technologies, learn from Germany’s mechanism in “policy and technology coordination”, improve the connection between “dual carbon” policies and digital economy policies, avoid policy fragmentation, achieve deep integration of digital economy and green development, and actively play a leading role in promoting green development through digital economy.

## Figures and Tables

**Table 1:** Evaluation system of green economic development level indicators.

Tier 1 Indicators	Secondary Indicators	Original Indicators	Indicator Properties
Green Innovation	Innovation Environment	Human capital index	+
		Number of R&D personnel	+
		Technology market turnover	+
	Innovation Input	R&D expenditure	+
		Science and technology expenditure accounts for local general public budget expenditure	+
		The proportion of eco-friendly expenditure to fiscal expenditure	+
	Innovation Output	Number of invention patents granted	+
		High-tech industry new product sales revenue accounted for the proportion of GDP	+
Green Sharing	Green Production	Total investment in environmental pollution control	+
		New product output value of industrial enterprises above the scale	+
		Electricity consumption per unit of gross regional product	−
	Green Living	Daily domestic water consumption per capita	−
		Green space coverage of built-up areas	+
		Harmless disposal rate of domestic waste	+

**Table 2:** Evaluation system of digital economy development level indicators.

Tier 1 Indicators	Secondary Indicators	Tertiary Indicators	Original Indicators	Indicator Properties
Digital economy development level	Digital Economy Development Scale	Digital Infrastructure	Long distance fiber optic cable line length	+
			Internet broadband access port	+
			Local switch capacity	+
		Digital Economy Popularization	Cell phone penetration rate	+
			Internet penetration rate	+
			Number of Internet domain names	+
		Digital Users	Number of people with Internet access	+
			Cell phone ownership per 100 households at the end of the year	+
			Number of digital TV subscribers	+
	Digital Technology Innovation	Digital Talent	Number of employees in the information service industry	+
		Digital Technology Investment	R&D expenditure of industrial enterprises above the scale	+
			The number of R&D projects (topics) of industrial enterprises above the scale	+
			Number of patent applications	+
	Digital Industry Development	Digital Trading	Telecommunications business volume	+
			E-commerce sales	+
			Courier volume	+
		Level of External Openness	Software Business Export	+
	Digital Finance	Coverage	Digital Financial Coverage	+
		Depth of Use	Depth of digital finance usage	+
		Degree of Digitization	Digitization of digital finance	+
	Digital Economy Development Potential	Government Support	Local financial science and technology expenditure	+
		Education Level	Number of full-time teachers in general higher education institutions	+
			Education Funding	+
		Enterprise Information Level	Number of corporate owned websites	+
			The proportion of enterprises with e-commerce trading activities	+

**Table 3:** Data element allocation level indicator rating system.

Tier 1 Indicators	Secondary Indicators	Original Indicators
Data Element Allocation Level	Data Element Management	Number of general higher education schools
		The equivalent full-time equivalent of R&D activity personnel of high-tech industry enterprises above the scale—person years
		New product development projects for high-tech industry enterprises above the scale
	Data Development Applications	New product development expenditure of high-tech industry enterprises above the scale
		New product sales revenue of high-tech industry enterprises above the scale
		Information technology service revenue
	Data Dissemination and Sharing	Number of pages
		Total postal business
		Number of websites
	Data Application Environment	Post Office Locations
		Total public library collections
		Broadcast program integrated population coverage

**Table 4:** Results of descriptive statistics of variables.

	Variables	Number of Observations	Average Value	Standard Deviation	Minimum Value	Maximum Value
Explained variables	Green Innovation	360	0.1359	0.1366	0.0137	0.7510
	Green Sharing	360	0.2007	0.1168	0.0544	0.8179
Explanatory variables	Digital Economy	360	0.1164	0.1048	0.0087	0.6719
Intermediate variables	Data Element Configuration	360	0.2590	0.1115	0.1050	0.7583
Control variables	Level of Government Intervention	360	0.5956	0.1205	0.3496	0.8960
	Level of Urbanization	360	0.0044	0.0040	0.0007	0.0250
	Human Capital Input	360	0.0568	0.0514	0.0112	0.2901
	Infrastructure Level	360	0.0201	0.0202	0.0000	0.1210
	Level of External Openness	360	0.0889	0.1177	0.0008	0.8116

**Table 5:** Baseline regression results for the impact of the digital economy on the green development of the economy.

Variables	(1) Env	(2) Eng	(3) Env	(4) Eng
Dig	1.014 *** (0.150)	1.048 *** (0.272)	0.580 *** (0.142)	0.801 *** (0.251)
Gov			0.152 ** (0.0714)	0.117 (0.120)
Urban			0.0852 (0.160)	0.543 (0.321)
Rz			28.03 *** (3.895)	12.51 (9.916)
Lnf			−0.0208 (0.0799)	−0.0576 (0.136)
Fdi			0.343 (0.261)	0.730 * (0.408)
Constant term	0.0178 (0.0120)	0.0793 *** (0.0205)	−0.133 (0.0918)	−0.279 (0.187)
Time/Individual	Yes	Yes	Yes	Yes
Observations	360	360	360	360
$R^{2}$	0.782	0.606	0.897	0.667

Note: The t-values adjusted for clustering robust standard errors are provided in parentheses; *, ** and *** represent significance at the 10%, 5%, and 1% levels, respectively.

**Table 6:** The dynamic impact of the digital economy on the green development of the economy.

Variables	(1) Env	(2) Eng	(3) Env	(4) Eng
l2.Dig	0.487 *** (0.135)	0.714 ** (0.279)		
l3.Dig			0.641 *** (0.203)	0.999 ** (0.409)
Constant term	−0.122 (0.116)	−0.322 (0.229)	−0.0179 (0.148)	−0.268 (0.269)
Control variables	Control	Control	Control	Control
Time/Individual	Yes	Yes	Yes	Yes
Observations	300	300	270	270
$R^{2}$	0.876	0.593	0.873	0.586

Note: The t-values adjusted for clustering robust standard errors are provided in parentheses; ** and *** represent significance at the 5%, and 1% levels, respectively.

**Table 7:** Robustness test and endogeneity treatment results.

Variables	Replacement of the Explanatory Variable Estimation Method		Shrinkage Processing		Instrumental Variables Method	
	Env	Eng	Env	Eng	Env	Eng
Dig	3.405 *** (0.778)	1.360 * (0.784)	0.492 *** (0.111)	0.637 *** (0.196)	0.670 *** (0.194)	1.160 *** (0.339)
Constant term	−0.986 * (0.545)	1.145 (0.902)	−0.112 (0.0983)	−0.309 (0.204)		
Phase I F-value					12.26	12.26
Hausmann Inspection					0.000	0.000
Control variables	Control	Control	Control	Control	Control	Control
Time/Individual	Yes	Yes	Yes	Yes	Yes	Yes
Observations	360	360	360	360	360	360
$R^{2}$	0.871	0.770	0.897	0.669	0.880	0.591

Note: The t-values adjusted for clustering robust standard errors are provided in parentheses; * and *** represent significance at the 10% and 1% levels, respectively.

**Table 8:** Results of intermediate effect test.

Variables	(1) Env	(2) Eng	(3) Ele	(4) Env	(5) Eng
Ele				0.665 *** (0.059)	0.953 *** (0.141)
Dig	0.580 *** (0.142)	0.801 *** (0.251)	0.617 *** (0.043)	0.118 ** (0.044)	0.139 (0.116)
Constant term	−0.133 (0.0918)	−0.279 (0.187)	0.002 (0.067)	−0.170 *** (0.054)	−0.332 (0.196)
Control variables	Control	Control	Control	Control	Control
Time/Individual	Yes	Yes	Yes	Yes	Yes
Observations	360	360	360	360	360
$R^{2}$	0.897	0.667	0.823	0.945	0.758

Note: The t-values adjusted for clustering robust standard errors are provided in parentheses; ** and *** represent significance at the 5% and 1% levels, respectively.

**Table 9:** Threshold test results.

Threshold Variables	Explained Variables	Number of Thresholds	F-Value	*p*-Value	Threshold Value			Threshold Value
					1%	5%	10%	
Dig	Env	Single Threshold	87.13	0.0000	39.8873	29.7561	24.4372	0.3797
	Eng	Single Threshold	85.40	0.0000	38.7340	27.4282	22.0718	0.3797
Reg	Env	Double Threshold	15.00	0.2010	104.9252	35.5007	19.6093	0.1125
			87.13	0.0000	36.7172	26.5059	23.3344	0.1173
	Eng	Double Threshold	15.66	0.2400	96.8097	33.0322	21.0822	0.3329
			85.40	0.0000	36.1849	25.5493	20.6971	0.3479

**Table 10:** Threshold regression results.

Variables	Threshold Variables			
Threshold Value	Dig		Reg	
	Env	Eng	Env	Eng
$Dig•I(Th\leq \theta_{1})$	0.096 (0.115)	0.979 *** (0.260)	0.096 (0.115)	0.979 *** (0.260)
$Dig•I(\theta_{1}<Th\leq \theta_{2})$	0.228 ** (0.090)	0.382 ** (0.139)	0.228 ** (0.090)	0.382 ** (0.139)
$Dig•I(Th>\theta_{2})$			0.339 *** (0.104)	0.509 *** (0.125)
Control variables	Control	Control	Control	Control
Number of periods	0.096 (0.115)	0.979 *** (0.260)	0.096 (0.115)	0.979 *** (0.260)
$R^{2}$	0.228 ** (0.090)	0.382 ** (0.139)	0.228 ** (0.090)	0.382 ** (0.139)

Note: ** and *** represent significance at the 5% and 1% levels, respectively.

**Table 11:** Results of regional heterogeneity test.

Variables	East		Medium		West	
	Env	Eng	Env	Eng	Env	Eng
Dig	0.867 *** (0.170)	1.209 *** (0.316)	0.178 ** (0.065)	0.130 (0.213)	0.189 (0.167)	0.217 * (0.102)
Constant term	−0.114 (0.133)	−0.444 (0.329)	−0.179 (0.174)	−0.290 (0.425)	−0.096 (0.136)	−0.454 (0.271)
Control variables	Control	Control	Control	Control	Control	Control
Time/Individual	Yes	Yes	Yes	Yes	Yes	Yes
$R^{2}$	0.940	0.772	0.922	0.516	0.724	0.488

Note: *, ** and *** represent significance at the 10%, 5%, and 1% levels, respectively.

**Table 12:** Results of the test for policy heterogeneity.

Variables	Areas Along the Route		Non-Frontline Areas		Pilot Areas		Non-Pilot Areas	
	Env	Eng	Env	Eng	Env	Eng	Env	Eng
Dig	0.434 ** (0.186)	0.566 * (0.323)	0.428 *** (0.130)	0.604 ** (0.231)	0.474 (0.343)	0.902 (0.757)	0.142 (0.092)	0.095 (0.087)
Constant term	0.017 (0.185)	−0.014 (0.251)	−0.155 * (0.079)	−0.622 * (0.307)	0.024 (0.468)	−1.623 * (0.646)	−0.148 (0.131)	0.068 (0.165)
Control Variables	Control	Control	Control	Control	Control	Control	Control	Control
Time/ Individuals	Yes	Yes	Yes	Yes	Yes	Yes	Yes	Yes
$R^{2}$	0.865	0.667	0.927	0.612	0.910	0.713	0.829	0.504

Note: *, ** and *** represent significance at the 10%, 5%, and 1% levels, respectively.

## Data Availability

Data is available on request from the author.
